# High-fat diet promotes renal injury by inducing oxidative stress and mitochondrial dysfunction

**DOI:** 10.1038/s41419-020-03122-4

**Published:** 2020-10-24

**Authors:** Yue Sun, Xin Ge, Xue Li, Jinrong He, Xinzhi Wei, Jie Du, Jian Sun, Xin Li, Zhe Xun, Weicheng Liu, Hao Zhang, Zhan-You Wang, Yan Chun Li

**Affiliations:** 1 Institute of Health Sciences, China Medical University, Shenyang, Liaoning China; 2 Department of Medicine, Division of Biological Sciences, The University of Chicago, Chicago, IL USA; 3 Department of Nephrology and Rheumatology, The Third Xiangya Hospital, Central South University, Changsha, Hunan China

**Keywords:** Apoptosis, Kidney

## Abstract

Obesity has been recognized as a major risk factor for chronic kidney disease, but the underlying mechanism remains elusive. Here, we investigated the mechanism whereby long-term high-fat diet (HFD) feeding induces renal injury in mice. The C57BL/6 mice fed HFD for 16 weeks developed obesity, diabetes, and kidney dysfunction manifested by albuminuria and blood accumulation of BUN and creatinine. The HFD-fed kidney showed marked glomerular and tubular injuries, including prominent defects in the glomerular filtration barrier and increased tubular cell apoptosis. Mechanistically, HFD feeding markedly increased triglyceride and cholesterol contents in the kidney and activated lipogenic pathways for cholesterol and triglyceride synthesis. HFD feeding also increased oxidative stress and induced mitochondrial fission in tubular cells, thereby activating the pro-apoptotic pathway. In HK-2 and mesangial cell cultures, high glucose, fatty acid, and TNF-α combination was able to activate the lipogenic pathways, increase oxidative stress, promote mitochondrial fission, and activate the pro-apoptotic pathway, all of which could be attenuated by an inhibitor that depleted reactive oxygen species. Taken together, these observations suggest that long-term HFD feeding causes kidney injury at least in part as a result of tissue lipid accumulation, increased oxidative stress, and mitochondrial dysfunction, which promote excess programmed cell death.

## Introduction

In the age of worldwide obesity pandemic, obesity has been recognized as a major and independent risk factor for the development of chronic kidney disease (CKD) and end-stage renal disease^1,2^. Obesity is a core manifestation of metabolic syndrome, which is characterized by the concurrent existence of hyperglycemia, diabetes, dyslipidemia, and cardiovascular disease^3^. In an obese state, the enlarged white adipose tissue not only serves to store triglycerides but also secrets a large amount of adipokines and pro-inflammatory cytokines and chemokines, which make obesity a chronic inflammation state. Chronic inflammation contributes to the development of insulin resistance^4^. Also contributing to insulin resistance is increased lipolysis in the adipose tissue under obesity that releases a large amount of free fatty acids (FFAs) into the bloodstream, which influx into peripheral tissues such as the liver and skeletal muscle, causing lipotoxicity to the tissues^5^. Tissue lipotoxicity induces mitochondrial dysfunction and promotes oxidative stress and endoplasmic reticulum stress^6^. In chronic a hyperlipidemic state, excess lipid accumulation also occurs in the kidney, and lipotoxicity drives the activation of pro-inflammatory, pro-fibrogenic, and pro-apoptotic pathways, causing cellular injury and renal dysfunction^7–9^. Long-term high-fat-diet (HFD) feeding, which results in obesity and produces metabolic syndrome, has been shown to alter renal lipid metabolism and induce lysosomal dysfunction leading to renal injury in mice^10,11^.

Mitochondria play an essential role in cellular metabolism and energy production that are required for all cellular functions. Mitochondria are the primary source of intracellular reactive oxygen species (ROS) production and contain a self-destructive arsenal of apoptogenic factors that can be unleashed to promote programmed cell death^12^. It is now well recognized that mitochondria are a dynamic subcellular organelle that is undergoing constant fusion and fission in response to metabolic and environmental stresses (such as excess nutrient intakes)^13^. Mitochondrial fusion and fission play critical roles in maintaining mitochondrial functions. Fusion helps to mitigate stress by mixing the contents of partially damaged mitochondria, whereas fission is needed to create new mitochondria. On the other hand, fission also contributes to quality control by removing damaged mitochondria and facilitating apoptosis during high levels of cellular stress. Fission is mediated by a cytosolic dynamin family member dynamin-related protein 1 (Drp1), which is recruited from the cytosol to form spirals around mitochondria that constrict to sever both inner and outer membranes^14,15^. The loss of mitochondrial membranes or increased mitochondrial membrane permeability in mitochondrial fission releases cytochrome c into cytoplasm, where cytochrome c binds to apoptotic protease-activating factor 1 (Apaf-1) to form apoptosome, which activates downstream caspases^16,17^. Drp1 is regulated by multiple posttranslational modifications (such as phosphorylation) that are induced by increases in intracellular Ca^++^ or ROS^18^. Previous studies have linked ROS and mitochondrial dysfunction to renal injury in experimental models of both acute kidney injury and CKD^19–23^.

Chronic hyperglycemia and hyperlipidemia associated with obesity drive excess nutrient influx to the kidney. We have recently reported that kidney injury occurs in nutrient overload as a result of ectopic lipid accumulation and epigenetic activation of pro-lipogenic and pro-fibrotic pathways within the kidney^7^. In this report, we presented evidence that HFD feeding promotes renal injury by inducing oxidative stress and mitochondrial dysfunction that lead to excess renal tubular apoptosis.

## Results

### Long-term HFD feeding induces obesity and metabolic syndrome

**Fig. 1:**
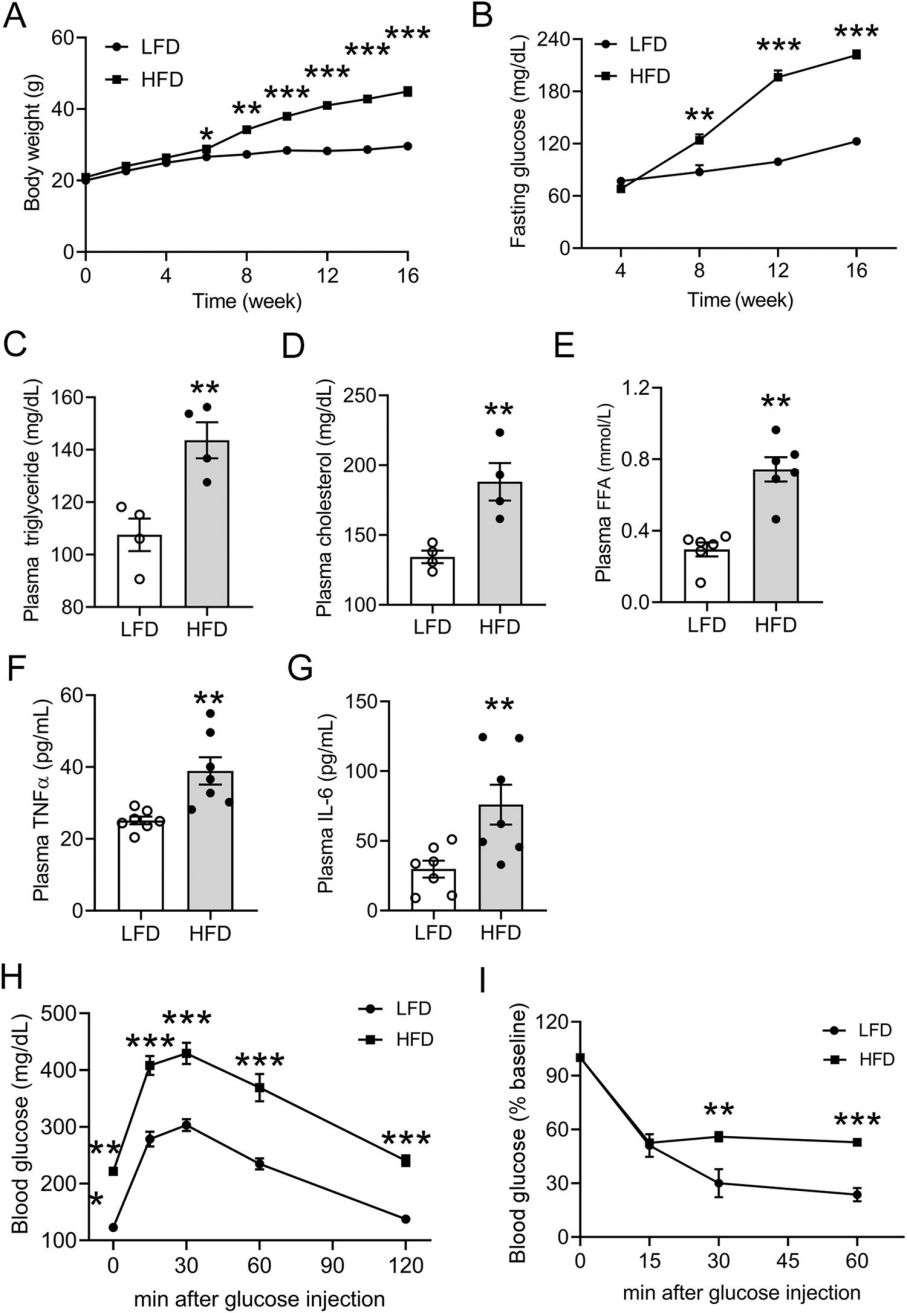
Long-term high-fat diet (HFD) feeding induces obesity and metabolic syndrome in the C57BL/6 mice. **A** Body-weight changes in 16 weeks of HFD or low-fat diet (LFD) feeding; **P < 0.01, ***P < 0.001 versus LFD, *n* = 5 each group. **B** Changes in fasting blood glucose in these mice. **C** Plasma triglyceride; **D** plasma cholesterol; **E** plasma-free fatty acids (FFA) at the end of 16 weeks. **P < 0.01 versus LFD; *n* = 4–6 each group. **F** Plasma TNF-α. **G** Plasma IL-6 at the end of 16 weeks. **P < 0.01 versus LFD, *n* = 7 each group. **H** Glucose tolerance test (GTT). **I** Insulin tolerance (ITT) at the end of 16 weeks. **P < 0.01, ***P < 0.001 versus LFD, *n* = 5 each group. Data in (**A**–**D**, **H**, and **I**) are representative of two independent experiments.

To investigate the effect of HFD-induced obesity on the kidney, we fed C57BL/6 mice an HFD for 16 weeks. As shown in Fig. 1, the mice that were fed the HFD showed a higher rate of body-weight gain compared to LFD-fed mice (Fig. 1A), and their body weight exceeded 40 g after 16 weeks of feeding. These HFD-fed mice developed time-dependent hyperglycemia (Fig. 1B). At 16 weeks, these mice exhibited marked increases in plasma concentrations of triglyceride, cholesterol, and FFA (Fig. 1C–E), and their plasma TNF-α and IL-6 levels were also significantly higher than the LFD counterparts (Fig. 1F, G), indicating the development of chronic systemic inflammation. Moreover, glucose tolerance test (GTT) and insulin tolerance test (ITT) showed that the HFD-treated mice developed severe glucose intolerance and insulin intolerance at the end of 16 weeks (Fig. 1H, I). These data demonstrated the establishment of an HFD-induced obese mouse model with severe metabolic syndrome.

### HFD induces lipid accumulation in the kidney

**Fig. 2:**
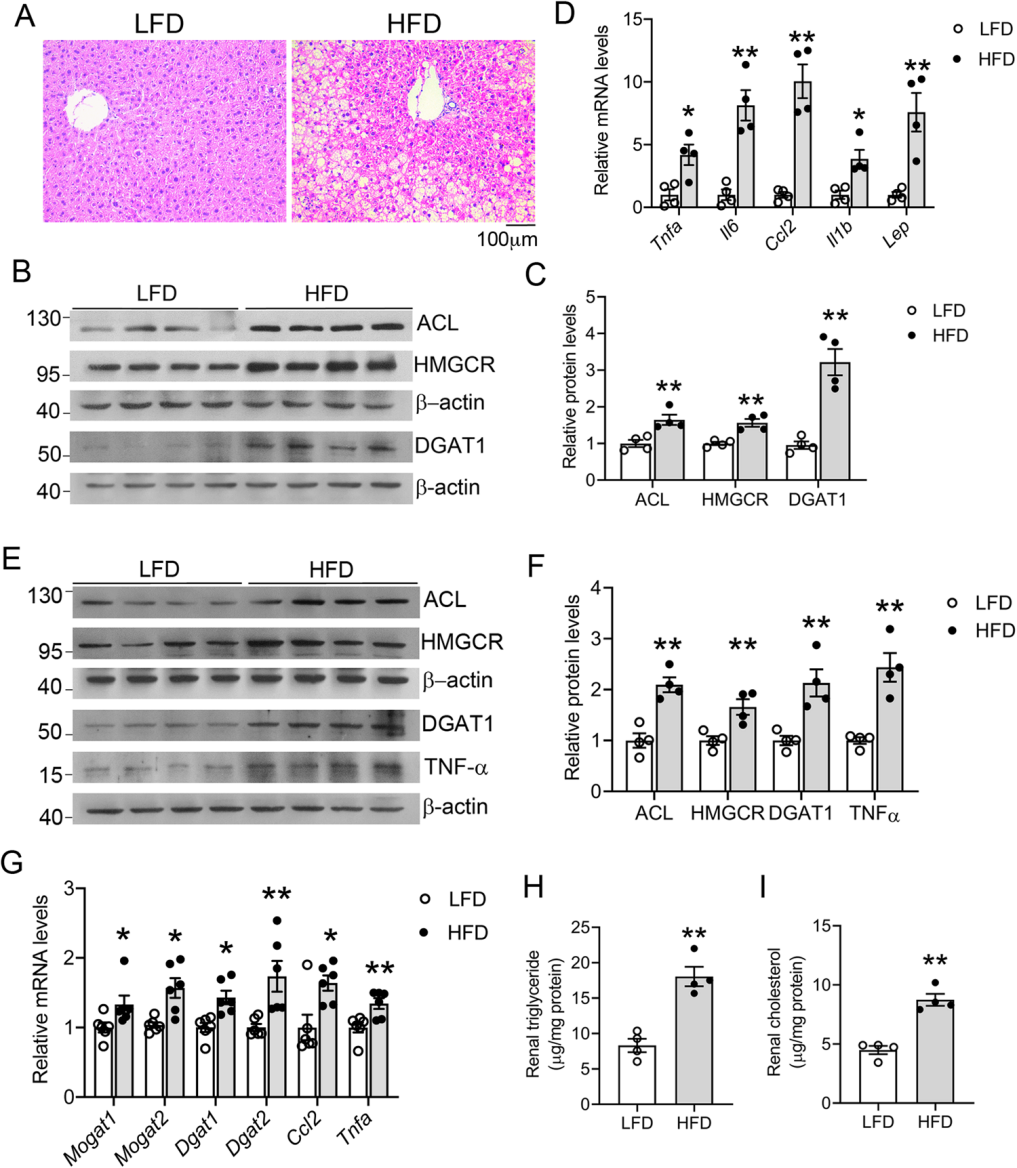
Long-term high-fat diet (HFD) feeding increases lipid accumulation in the liver and kidney. **A** Representative H&E stained liver sections (magnification 200x) from LFD- and HFD-fed mice for 16 weeks. **B**, **C** Western blots (**B**) and densitometric quantitation (**C**) of indicated proteins in liver lysates from LFD- or HFD-fed mice; **P < 0.01 versus LFD, *n* = 4 each group. **D** Quantitative real-time PCR analysis of TNF-α, IL-6, Ccl2, IL-1β, and leptin transcripts in the liver of LFD- or HFD-fed mice; *P < 0.05, **P < 0.01 versus LFD, *n* = 4 each group. **E**, **F** Western blots (**E**) and densitometric quantitation (**F**) of indicated proteins in the abdominal fat of LFD- and HFD-fed mice; **P < 0.01 versus LFD, *n* = 4 each group. **F** Quantitative real-time PCR data for the transcripts of MOGAT1, MOGAT2, DGAT1, DGAT2, Ccl2, and TNF-α in the kidneys of LFD- or HFD-fed mice. *P < 0.05, **P < 0.01 versus LFD, *n* = 4 each group. **H** Renal triglyceride. **I** Renal cholesterol in the kidney cortex at the end of 16 weeks. **P < 0.01 versus LFD; *n* = 4 each group. Data in (**A**–**C**, **E**, **F**, **H**, and **I**) are representative of two independent experiments.

As expected, the HFD-fed mice developed fatty liver at 16 weeks (Fig. 2A). Western blot analyses showed that the expression of ATP-citrate lyase (ACL), HMG-CoA reductase (HMGCR), and diacylglycerol O-acyltransferase 1 (DGAT1) was stimulated in the liver (Fig. 2B, C). ACL is an enzyme that converts citrate to acetyl-CoA, the common building block for de novo lipogenesis, HMGCR is the rate-limiting enzyme in the cholesterol biosynthesis pathway, and DGAT1 catalyzes the final step of triglyceride formation. Therefore, the lipogenic pathways were activated in the HFD-fed liver. Moreover, the expression of pro-inflammatory cytokines and chemokine (*Tnfa, Il6, Ccl2*, and *Il1b*) and leptin were also induced in the abdominal adipose tissue (Fig. 2D), consistent with the chronic systemic inflammatory status of these mice. Interestingly, we also detected marked upregulation of ACL, HMGCR, DGAT1, and TNF-α in the kidney cortex of HFD-fed mice at the end of 16 weeks (Fig. 2E, F), and real-time PCR quantitation confirmed the increases in *Tnfa, Ccl2, Dgat1*, and *Dgat2* transcripts, as well as those of monoacylglycerol O-acyltransferase 1 and 2 (*Mogat1* and *Mogat2*) in the kidney cortex (Fig. 2G), indicating that, in addition to the liver, the lipogenic pathways were also stimulated in the kidney under long-term HFD feeding. Consistent with this notion, quantitation of lipids extracted from the kidney showed marked increases in triglyceride and cholesterol contents in the kidney of HFD-treated mice (Fig. 2H, I). It is conceivable that this ectopic lipid accumulation in the kidney causes lipotoxicity leading to renal injury.

### HFD-fed mice develop severe nephropathy

**Fig. 3:**
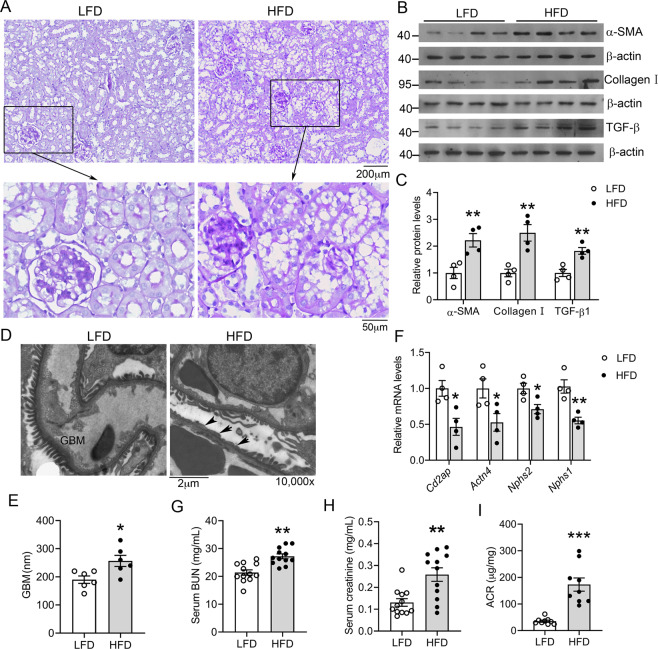
Long-term high-fat diet (HFD) feeding induces renal injury. **A** Periodic acid-Schiff (PAS)-stained kidney sections from LFD- or HFD-fed mice at the end of 16 weeks. Lower panes are enlarged images of the boxed areas. **B**, **C** Western blots (**B**) and densitometric quantitation (**C**) of indicated proteins in kidney lysates from HFD- or LFD-fed mice; **P < 0.01 versus LFD, *n* = 4 each group. Data in (**A**–**C**) are representative of two independent experiments. **D** Transmission electron microscopic examination of the glomerular filtration barrier of LFD- or HFD-treated mice. GMB, glomerular basement membrane. Arrows indicate areas of podocyte foot-process effacement. **E** GMB thickness in LFD- or HFD-fed mice; *P < 0.05 versus LFD, *n* = 6 each group. **F** Quantitative real-time PCR quantitation of mRNA levels of CD2AP, α-Actinin-4, podocin, and nephrin in the renal cortex. *P < 0.05, **P < 0.01 versus LFD, *n* = 4 each group. **G** Serum BUN; **H** serum creatinine; **I** urinary albumin to creatinine ratio (ACR) in LFD- or HFD-fed mice at the end of 16 weeks. **P < 0.01, ***P < 0.001 versus LFD, *n* = 9–12 each group.

Histological examination revealed the severe renal injury in the mice after 16 weeks of HFD feeding. As shown in Fig. 3, periodic acid-Schiff (PAS) staining demonstrated various degrees of glomerulosclerosis and remarkable tubular injury in HFD-fed mice (Fig. 3A). The tubular area of HFD-fed mice, especially the proximal convoluted tubule, showed severe brush border disruption and epithelial cell shedding (Fig. 3A). Consistent with the glomerular fibrosis, western blot data showed increased expression of α-SMA, type 1 collagen, and TGF-β1 in the renal cortex of HFD-treated mice (Fig. 3B, C), indicating activation of the fibrogenic pathway. Electron microscopic examination revealed thickening of the glomerular basement membrane (GBM) and podocyte foot-process effacement in HFD-treated mice (Fig. 3D, E). The transcripts of key structural proteins forming the slit diaphragm, including CD2AP, α-actinin-4, podocin, and nephrin, were all reduced in HFD-fed mice (Fig. 3F). All these are signs of glomerular filtration barrier dysfunction. Furthermore, the HFD-fed mice exhibited marked and significant increases in blood BUN and creatinine concentrations (Fig. 3G, H) and in urinary albumin excretion (Fig. 3I), confirming that these mice had developed physiologically impaired renal function.

### HFD induces oxidative stress and mitochondrial dysfunction in the kidney

**Fig. 4:**
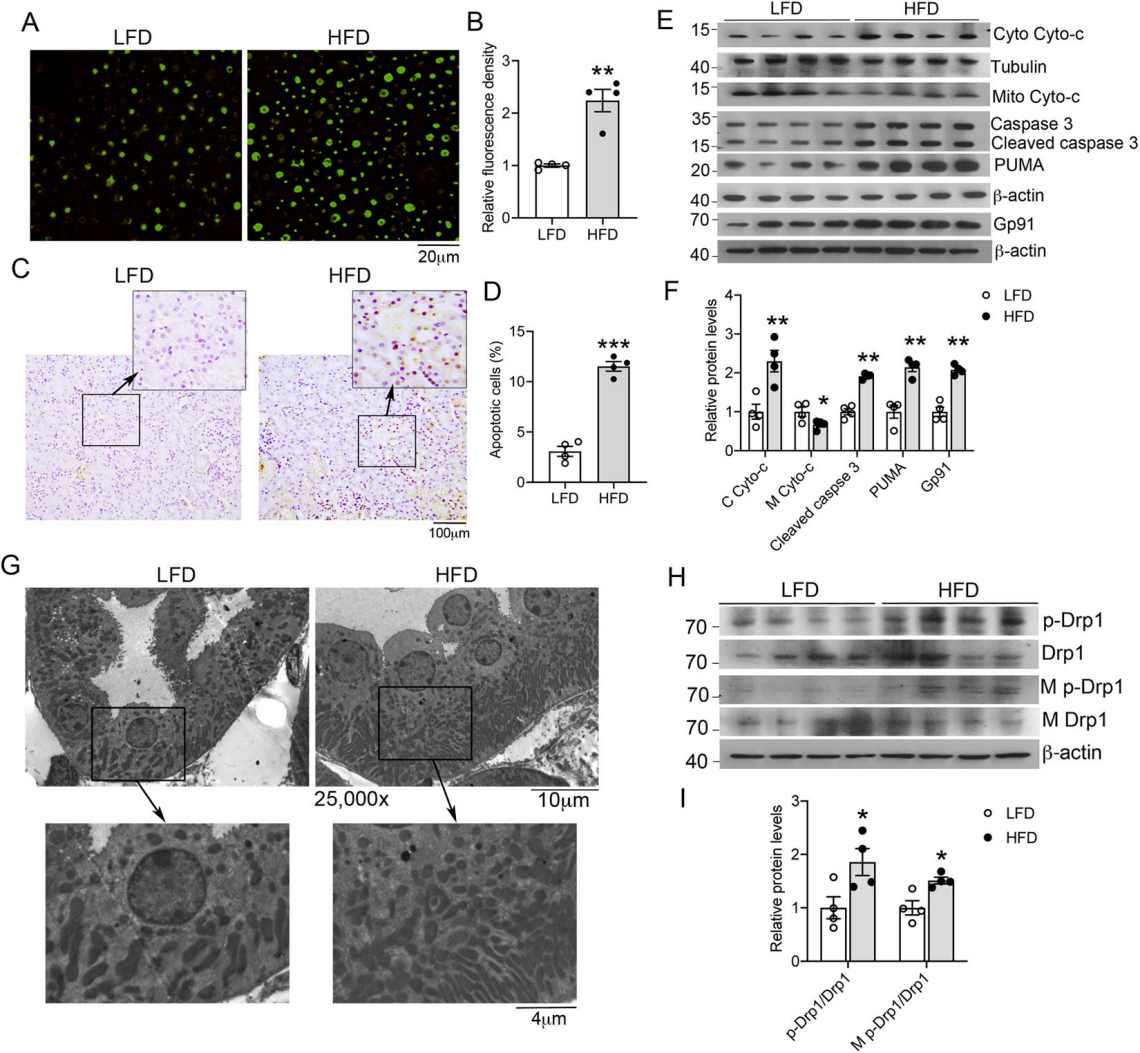
High-fat diet (HFD) feeding promotes oxidative stress and mitochondrial dysfunction in the kidney. **A** Fluorescence microscopic images of DCFH-DA staining of kidney cell suspension from LFD- or HFD-fed mice, magnification ×200. **B** Quantitation of fluorescence intensity of DCFH-DA stained cells. **P < 0.01 versus LFD, *n* = 4 each group. **C** TUNEL staining of kidney sections. Enlarged images are the boxed areas. **D** Semi-quantification of TUNEL stain-positive kidney cells. In each mouse kidney section, 20 random microscopic fields (×200 magnification) were counted for TUNEL-positive cells, and the data are expressed as average positive cells per field. ***P < 0.001 versus LFD, *n* = 4 each group. **E**, **F** Western blot analysis (**E**) of cytosolic (C) and mitochondrial (M) cytochrome (Cyto)-c, caspase-3, PUMA, and Gp91 in the kidney cortex and their densitometric quantitation (**F**); *P < 0.05, **P < 0.01 versus LFD, *n* = 4 each group. **G** Transmission electron microscopic examination of proximal tubular epithelial cells. The enlarged images are the boxed areas. **H** Protein levels of total cellular Drp1 and phospho-Drp1, and mitochondrial (M) Drp1 and phospho-Drp1 in the renal cortex of LFD- or HFD-fed mice. **I** Densitometric quantitation of phospho-Drp1 to Drp1 ratio in the total cell lysates and the mitochondrial fraction; *P < 0.05 versus LFD, *n* = 4 each group. Data in (**A**, **B**, **E**, **F**, **H**, and **I**) are representative of two independent experiments.

To explore the mechanism of renal injury caused by HFD feeding, we assessed renal levels of ROS by DCFH-DA staining. As shown in Fig. 4, 16 weeks of HFD feeding dramatically increased intracellular ROS generation in the kidney cortex cells (Fig. 4A, B). TUNEL staining revealed >threefold increases in renal tubular cell apoptosis in HFD-fed mice (Fig. 4C, D). At the molecular level, HFD feeding induced the expression of Gp91, a subunit of NADPH oxidase that usually serves as a marker of oxidative stress, and promoted cytochrome c release from mitochondria into the cytoplasm; meanwhile, the expression of p53 upregulated modulator of apoptosis (PUMA), a pro-apoptotic protein of Bcl-2 family, and caspase-3 activation were also highly stimulated (Fig. 4E, F), suggesting that cytochrome c release activated the pro-apoptotic pathway. Interestingly, electron microscopic analysis revealed a striking increase in mitochondrial numbers in the proximal tubular epithelial cells in the HFD-fed mouse kidney (Fig. 4G). These mitochondria were generally smaller compared with those in the LFD-treated mouse kidney, suggesting that constant mitochondrial fission occurred in renal tubular cells under HFD feeding. As Drp1 is a critical mediator of mitochondrial fission, we examined whether Drp1 is activated by HFD by measuring the levels of Drp1 and phospho-Drp1(S616) in kidney cortex lysates as well as in purified mitochondria. Western blot data showed that both total cellular phospho-Drp1 and mitochondria-associated phospho-Drp1 were substantially increased in the kidney cortex of HFD-fed mice (Fig. 4H, I), strongly suggesting that mitochondrial fission was involved in driving renal tubular cell apoptosis.

### Glucose, fatty acid, and cytokine induce oxidative stress and mitochondrial dysfunction in renal tubular cells

**Fig. 5:**
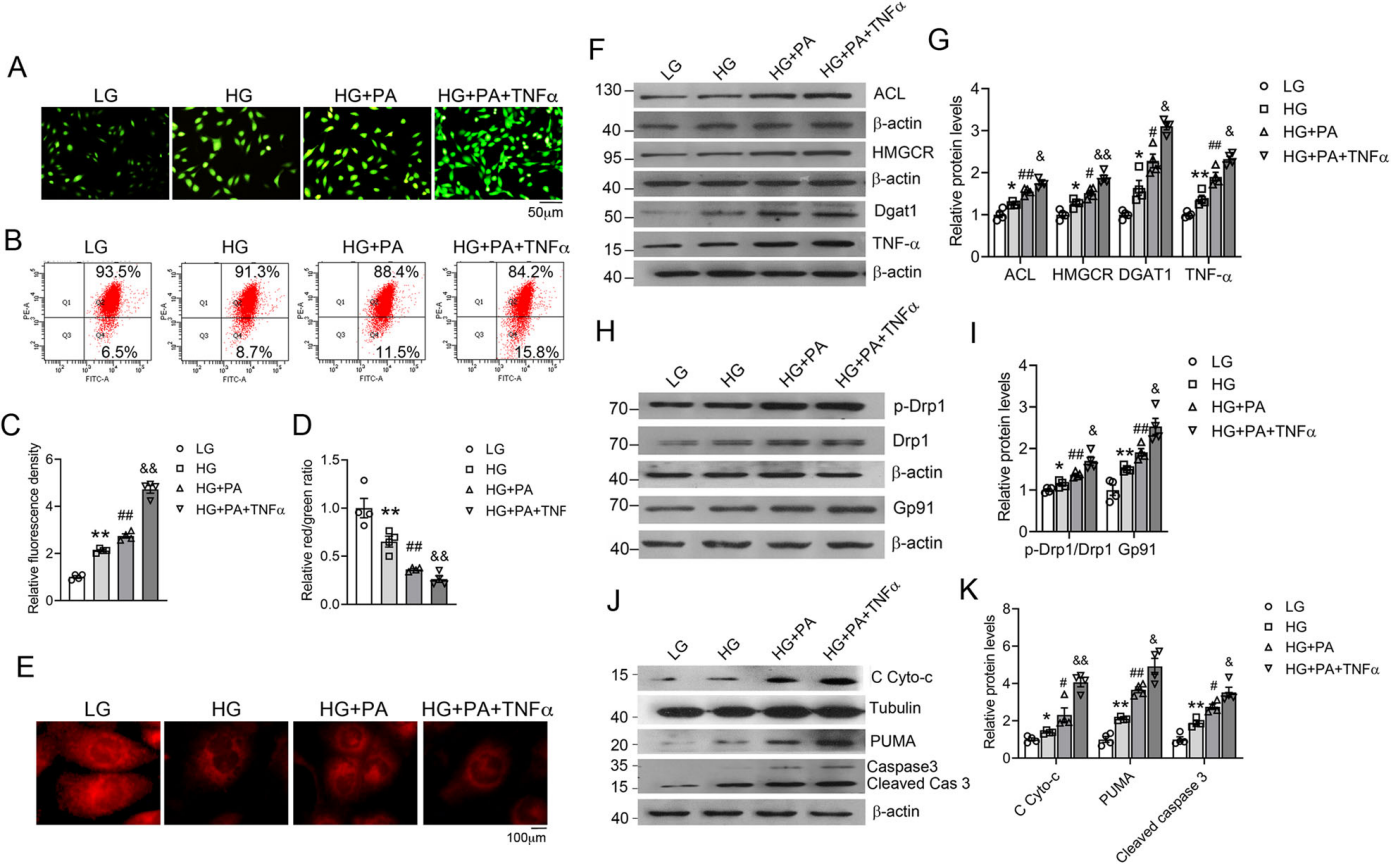
High glucose, fatty acid, and pro-inflammatory cytokine induce oxidative stress and mitochondrial dysfunction in HK-2 cells. **A** Representative images of reactive oxygen species (ROS)-induced fluorescence in DCFH-DA stained HK-2 cells cultured under different treatments as indicated (magnification, ×100). **B** Mitochondrial membrane potential of HK-2 cells under various treatments assessed by flow cytometry after JC-1 staining. **C** Quantitative ROS-induced fluorescence in HK-2 cells; **P < 0.01 versus LG; ^##^*P* < 0.01 versus HG; ^&&^*P* < 0.01 versus HG + PA; *n* = 4 each group. **D** Quantitation of mitochondrial membrane potential based on flow-cytometry analysis of JC-1-stained cells. *P < 0.05 versus LG; ^##^*P* < 0.01 versus HG; ^&&^*P* < 0.01 versus HG + PA; *n* = 4 each group. **E** Representative confocal microscopic images of HK-2 cells stained with MitoTracker Red. **F**, **G** Representative western blots (**F**) and densitometric quantitation (**G**) of ATP-citrate lyase (ACL), HMGCR, DGAT1, and TNF-α proteins in HK-2 cells with various treatments. **H**, **I** Representative western blots (**H**) and densitometric quantitation (**I**) of total cellular p-Drp1, Drp1, and Gp91 proteins. **J**, **K** Representative western blots (**J**) and densitometric quantitation (**K**) of cytosolic (C) Cyto-c, PUMA, and caspase 3. *P < 0.05, **P < 0.01 versus LG; ^#^*P* < 0.05, ^##^*P* < 0.01 versus HG; ^&^*P* < 0.05, ^&&^*P* < 0.01 versus HG + PA; *n* = 4 each group.

Since the HFD-fed mice developed hyperglycemia, hyperlipidemia with increased circulating FFA, and chronic inflammation with increased circulating pro-inflammatory cytokines, we speculated that these factors are involved in driving renal injury in these mice. To mimic these factors, we exposed HK-2 cells, a renal tubular cell line, to high glucose (HG), palmitic acid (PA), TNF-α, or their combination. PA was selected because it is a major long-chain saturated fatty acid that is known to alter cellular metabolism, increase oxidative stress, and promote inflammation^7,24^. As shown in Fig. 5, these factors, especially their combinations, were able to markedly increase ROS production (Fig. 5A, C) and disrupted mitochondrial membrane potential, as revealed by JC-1 assays (Fig. 5B, D). JC-1 dye exhibits potential-dependent accumulation in mitochondria, indicated by a fluorescence emission shift from green to red. Mitochondrial depolarization is indicated by a decrease in the red/green fluorescence intensity ratio. The combinations also disrupted mitochondrial morphology visualized by a mitochondrial fluorescent probe (MitoTracker Red CMXRos) (Fig. 5E). At the molecular level, the combinations were able to upregulate ACL, HMGCR, DGAT1, and TNF-α expression (Fig. 5F, G), indicating activation of the lipogenic pathways. The combinations were also able to induce Gp91 expression and Drp1 phosphorylation (Fig. 5H, I), drive cytochrome c release from mitochondria and activate the PUMA-caspase-3 pro-apoptotic pathway (Fig. 5J, K). In all these assays, the combination of the three factors had the maximal effect, suggesting that hyperglycemia, hyperlipidemia, and inflammation are the driving force for renal injury in the HFD-induced obesity model.

**Fig. 6:**
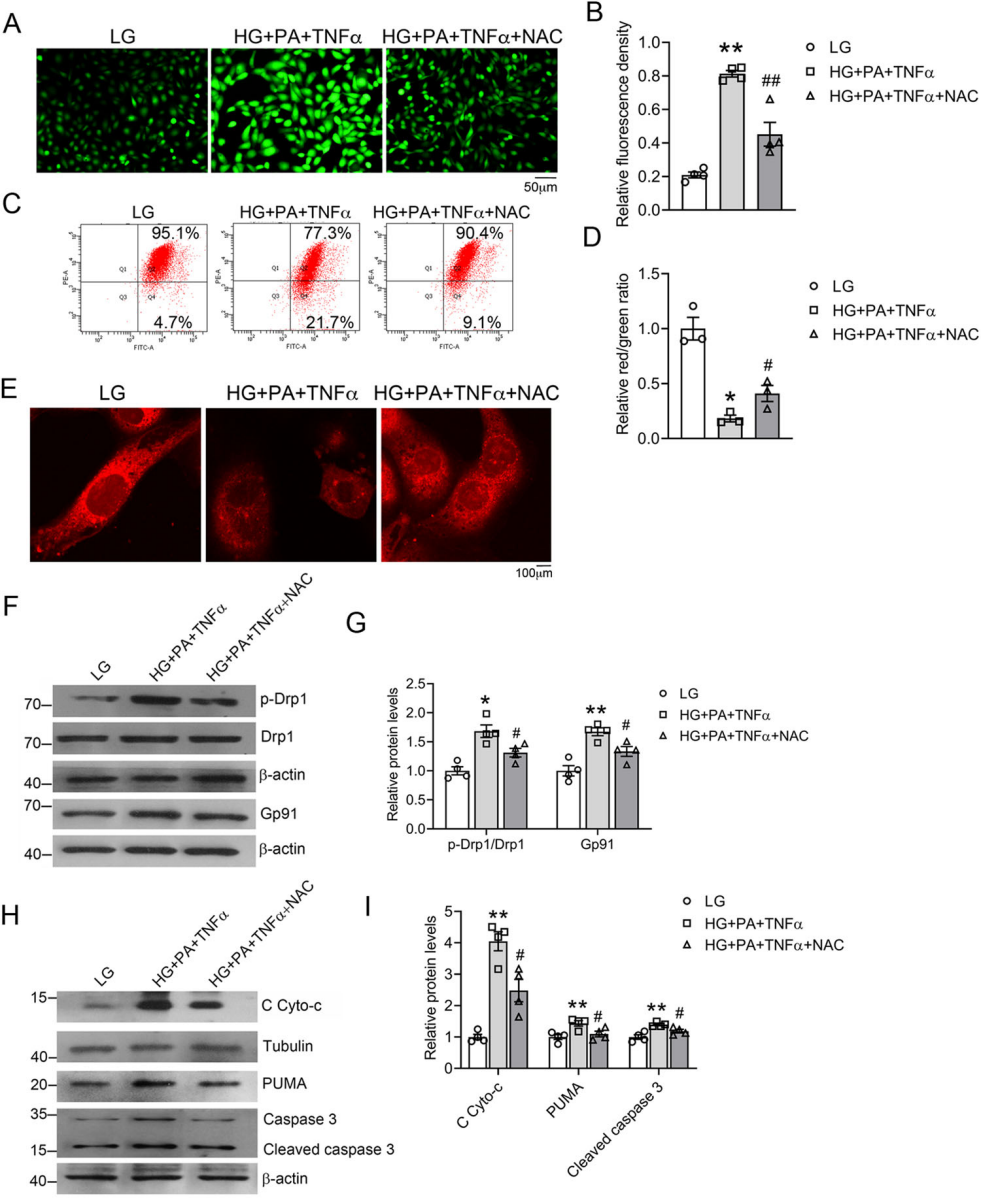
Reactive oxygen species (ROS) depletion by NAC treatment attenuates the effects of high glucose, fatty acid, and pro-inflammatory cytokine in HK-2 cells. **A**, **B** Representative images of ROS-induced fluorescence (**A**) and quantification (**B**) of ROS production in HK-2 cells under different treatments, as indicated (magnification, ×100). **P < 0.01 vs. LG; ^##^*P* < 0.01 vs. HG + PA + TNF, *n* = 4 each group. **C**, **D** Flow-cytometric analysis of mitochondrial membrane potential after JC-1 staining (**C**) and quantitation (**D**); *P < 0.05 vs. LG; ^#^*P* < 0.05 vs. HG + PA + TNF, *n* = 3 each group. **E** Confocal microscopic mitochondrial images in HK-2 cells stained with MitoTracker Red. **F**, **G** Representative western blots (**F**) and densitometric quantitation (**G**) of total cellular p-Drp1, Drp1, and Gp91 in HK-2 cells with different treatments, *P < 0.05, **P < 0.01 vs. LG; ^#^*P* < 0.05 vs. HG + PA + TNF; *n* = 4 each group. **H**, **I** Representative western blots (**H**) and quantitation (**I**) of cytosolic (C) Cyto-c, PUMA and caspase-3; *P < 0.05, **P < 0.01 vs. LG; ^#^*P* < 0.05 vs. HG + PA + TNF; *n* = 4 each group.

ROS is known to activate Drp1 and promote mitochondrial fission. To assess the detrimental effect of ROS in mitochondrial dysfunction and apoptosis, we exposed HK-2 cells to the HG, PA, and TNF-α combination in the presence or absence of NAC, an antioxidant that scavenges ROS inside the cell^25^. As shown in Fig. 6, NAC markedly blocked ROS production that was induced by the combination (Fig. 6A, B). NAC also effectively reduced mitochondrial depolarization (Fig. 6C, D) and prevented mitochondrial disruption (Fig. 6E). Western blot analyses showed that NAC treatment attenuated the induction of Gp91 and Drp1 phosphorylation (Fig. 6F, G), prevented the release of cytochrome c from mitochondria, and abrogated the induction of PUMA and caspase 3 (Fig. 6H, I). These observations indicate that depletion of ROS ameliorates mitochondrial dysfunction and inhibits renal cell apoptosis, confirming a critical role of ROS in HFD-induced renal injury.

**Fig. 7:**
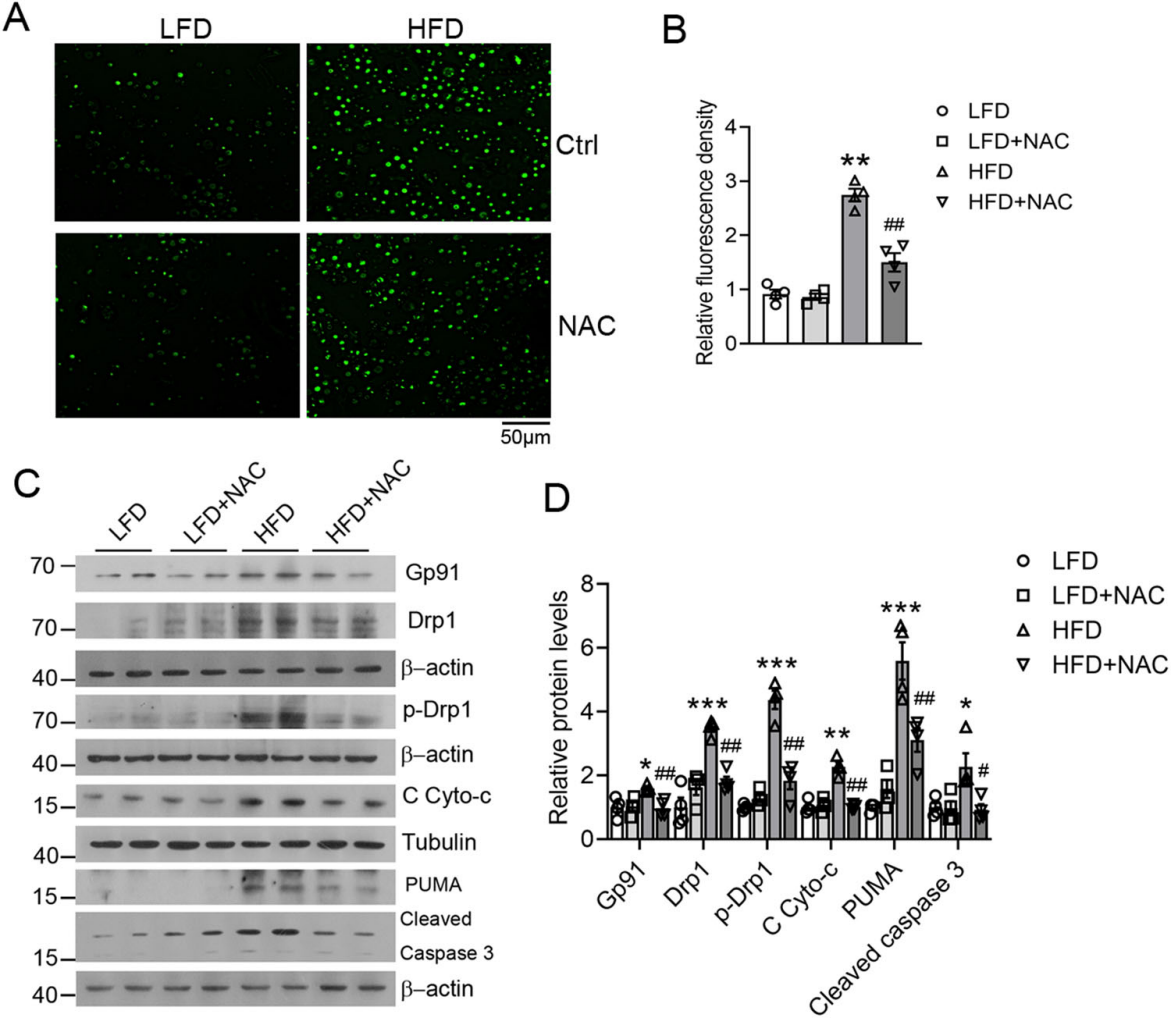
NAC treatment attenuates oxidative stress and mitochondrial dysfunction in kidney cortex cultures. **A**, **B** Representative images of reactive oxygen species (ROS)-induced fluorescence (**A**) and its quantification (**B**) in DCFH-DA-stained kidney cell suspension from LFD- or HFD-fed mice with or without NAC treatment. **P < 0.01 vs. LFD; ^##^*P* < 0.01 vs. HFD, *n* = 4 each group. **C**, **D** Representative western blots (**C**) and densitometric quantitation (**D**) of indicated proteins in kidney cortex cultures from LFD- or HFD-fed mice with or without NAC treatment; *P < 0.05, **P < 0.01, ***P < 0.001 vs. LFD; ^#^*P* < 0.05, ^##^*P* < 0.01 vs. HFD; *n* = 4 each group. C Cyto-c, cytosolic cytochrome c.

We next used an ex vivo kidney tissue culture system to confirm that the effect of NAC on mitochondrial dysfunction and apoptosis in the kidney. This ex vivo kidney tissue culture was reported to mimic the kidney in terms of pharmacological responses^7^. As shown in Fig. 7, the kidney cortex isolated from HFD-fed mice showed elevated ROS production compared with LFD-fed mice, and NAC treatment was able to significantly suppress the ROS levels (Fig. 7A, B). Importantly, NAC treatment was also able to block the induction of Gp91, Drp1 phosphorylation, cytochrome c release into the cytosol, and induction of PUMA and caspase 3 (Fig. 7C, D). These observations indicate that depletion of ROS by NAC can indeed attenuate oxidative stress, prevent mitochondrial dysfunction, and block cytochrome c-mediated apoptotic pathways in the kidney cortex.

### Glucose, fatty acid, and cytokine induce oxidative stress and mitochondrial dysfunction in mesangial cells (MCs)

**Fig. 8:**
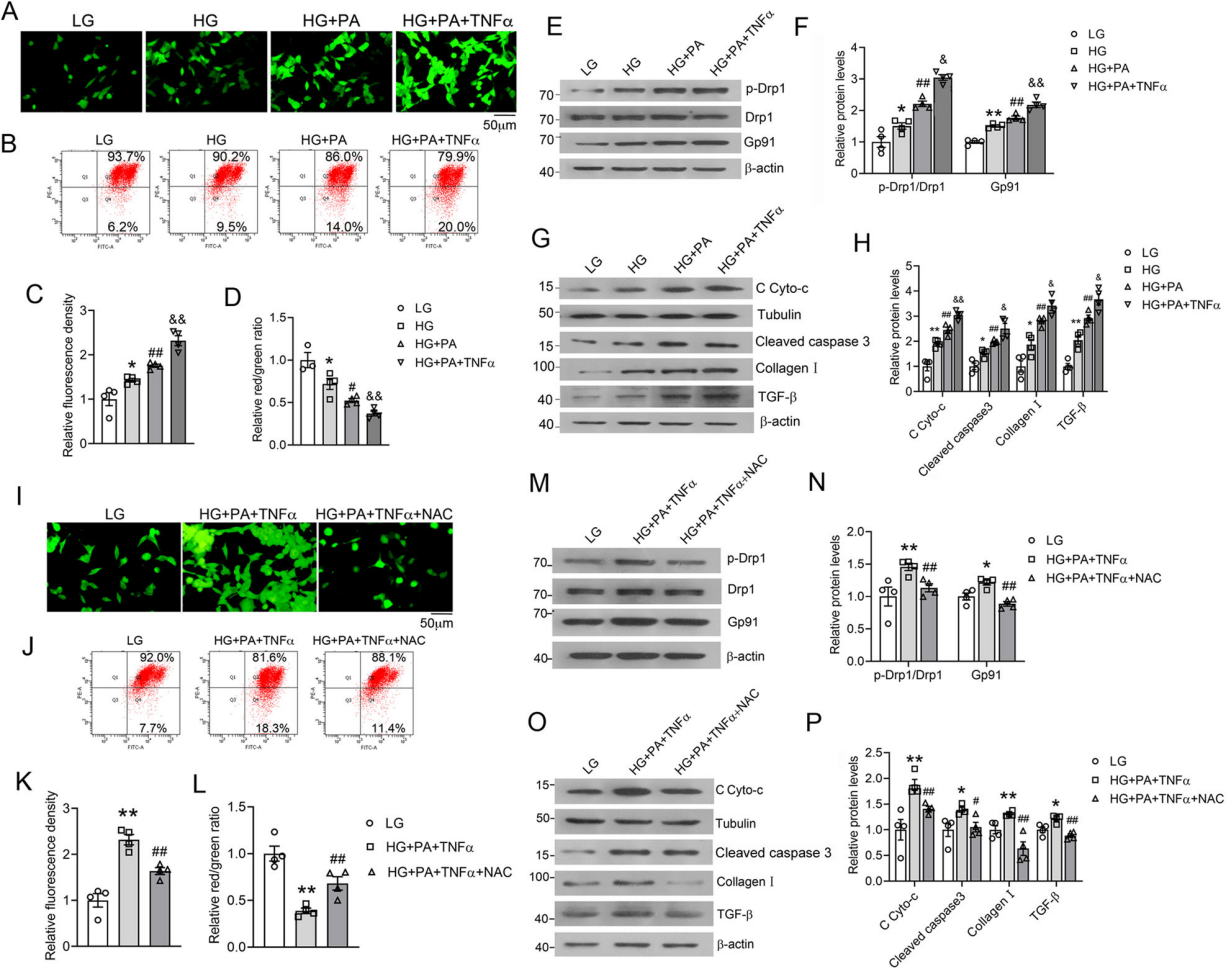
High glucose, fatty acid, and pro-inflammatory cytokine induce oxidative stress and mitochondrial dysfunction in mesangial cells (MCs). **A** Representative images of reactive oxygen species (ROS)-induced fluorescence in DCFH-DA-stained MCs cultured under different treatments as indicated (magnification, ×100). **B** Mitochondrial membrane potential of MCs assessed by flow cytometry after JC-1 staining. **C** Quantitative ROS-induced fluorescence. **D** Quantitation of mitochondrial membrane potential in JC-1-stained cells. *P < 0.05 versus LG; ^#^*P* < 0.05, ^##^*P* < 0.01 versus HG; ^&&^*P* < 0.01 versus HG + PA; *n* = 4 each group. **E**, **F** Representative western blots (**E**) and densitometric quantitation (**F**) of total p-Drp1, Drp1, and Gp91 proteins. **G**, **H** Representative western blots (**G**) and densitometric quantitation (**H**) of cytosolic (C) Cyto-c, caspase 3, type 1 collagen, and TGF-β proteins. *P < 0.05, **P < 0.01 versus LG; ^#^*P* < 0.05, ^##^*P* < 0.01 versus HG; ^&^*P* < 0.05, ^&&^*P* < 0.01 versus HG + PA; *n* = 4 each group. **I** Representative images of ROS-induced fluorescence in MCs with or without NAC treatment. **J** Flow-cytometric analysis of mitochondrial membrane potential in MCs with or without NAC treatment. **K** Quantification of ROS production in MCs in the presence or absence of NAC. **L** Quantification of mitochondrial membrane potential in MCs in the presence or absence of NAC. **P < 0.01 vs. LG; ^##^*P* < 0.01 vs. HG + PA + TNF; *n* = 4 in each group. **M**, **N** Representative western blots (**M**) and densitometric quantitation (**N**) of total p-Drp1, Drp1, and Gp91 proteins in MCs in the presence or absence of NAC; *P < 0.05, **P < 0.01 vs. LG; ^##^*P* < 0.01 vs. HG + PA + TNF; *n* = 4 each group. **O**, **P** Representative western blots (**O**) and quantitation (**P**) of cytosolic Cyto-c, caspase-3, type 1 collagen, and TGF-β; *P < 0.05, **P < 0.01 vs. LG; ^#^*P* < 0.05, ^##^*P* < 0.01 vs. HG + PA + TNF, *n* = 4 each group.

Glomerular MCs are the main contributor to glomerular damage; therefore, we further used MC cultures to test the effects of HG, PA, and TNF-α. Similarly, as seen in HK-2 cells, HG, PA, and TNF-α combination maximized the production of ROS in MCs (Fig. 8A, C) and had the greatest impact on mitochondrial membrane potential disruption (Fig. 8B, D). As expected, this combination markedly induced Gp91 expression and Drp1 phosphorylation (Fig. 8E, F), and promoted cytochrome c release and caspase-3 activation (Fig. 8G, H). It also stimulated TGF-β1 and type 1 collagen expression (Fig. 8G, H). However, the effects of HG, PA, and TNF-α combination were substantially attenuated by the presence of NAC. NAC blocked the production of ROS (Fig. 8I, K) and reversed the decrease in mitochondrial membrane potential (Fig. 8J, L). NAC also prevented the induction of Gp91, Drp1 phosphorylation, cytochrome c release, caspase-3 activation, and the upregulation of TGF-β1 and type 1 collagen (Fig. 8M–P). Together these observations strongly suggest that increased oxidative stress is a key event that leads to mitochondrial dysfunction and excess renal cell apoptosis under long-term HFD feeding.

## Discussion

In this study, we demonstrated that long-term HFD feeding can induce kidney injury in the C57BL/6 mice, and cellular oxidative stress and mitochondrial dysfunction play critical roles in mediating renal injury. Our data showed that 16-week HFD feeding induced obesity and diabetes in mice that exhibited high levels of circulating FFA and pro-inflammatory cytokines. Importantly, these HFD-fed mice also exhibited abnormal accumulation of lipids in the liver as well as in the kidney. Consistent with our previous reports^7^, kidney accumulation of lipids causes lipotoxicity that set the stage for renal injury. The lipotoxic effects that we observed in this study were reflected by heightened oxidative stress and active mitochondrial fission that trigger the activation of pro-apoptotic pathway leading to excess renal cell apoptosis. The manifestation of renal injury in the HFD-fed mice included glomerular fibrosis, podocyte foot-process effacement, and tubular cell apoptosis, and the pathological consequence was kidney malfunction and development of albuminuria.

There have been a number of prior studies that reported the detrimental effects of HFD on the kidney. For example, Kume et al. described that HFD feeding stimulates lipogenic enzymes in the fatty acid synthesis pathway but suppresses lipolysis, which subsequently drives the excess accumulation of lipids within the kidney. This altered lipid metabolism eventually causes renal injury, including glomerulosclerosis, interstitial fibrosis, and albuminuria^11^. Yamamoto et al. reported that HFD feeding results in phospholipid accumulation in lysosomes within renal proximal tubular cells that subsequently impairs autophagic flux leading to kidney injury^10^. Therefore, it appears that diets with high-fat contents have multiple ways to induce kidney injury.

In this study, we set to explore the mechanism whereby HFD promotes renal injury by focusing on mitochondrial dysfunction, which has been recognized as a critical driver for cell injury and cell death^18^. We reasoned that with the development of hyperglycemia and hyperlipidemia in HFD-fed mice, kidney cells are exposed to an excess amount of nutrients. Cellular metabolism would have to increase in response to the excess nutrient influx to the cells, and this metabolic stress would promote mitochondrial renewal and fission. Metabolic stress could also increase ROS production, a byproduct of electron transport on mitochondrial membranes and ROS, in turn, can further drive mitochondrial fission by activating Drp1^18^. One consequence of excess mitochondrial fission is cytochrome c release and subsequent activation of the pro-apoptotic pathway^16^. Indeed, in the kidney of HFD-fed mice, we did detect massive ROS production as well as active mitochondrial fission, best illustrated in the proximal tubular cells. At the molecular level, an oxidative stress marker Gp91 was highly induced in HFD kidney. Importantly, consistent with active mitochondrial fission, the HFD-fed kidney exhibited a dramatic increase in Drp1 phosphorylation as well as Drp1 recruitment to mitochondria. Similarly, HFD-induced Drp1 phosphorylation was previously reported in the brain^26^. Drp1 phosphorylation activates Drp1, and Drp1 recruitment to mitochondria is required for mitochondrial fission to take place^27^. Accompanying Drp1 activation and mitochondrial fission, cytochrome c release into the cytosol was also markedly elevated in the HFD kidney, which well explains the activation of caspase 3 and increased apoptosis seen in the HFD kidney. Together these observations support the conclusion that HFD-induced renal injury is at least in part caused by excessive oxidative stress and mitochondrial dysfunction. In support of this view, excess mitochondrial fission caused by Drp1 activation in tubular cells was also reported in mouse models of acute kidney injury^19^.

Our data from in vitro and ex vivo cultures support this conclusion. We demonstrated that exposure of tubular cells (HK-2) or mesangial cells to high glucose, palmitic acid, and/or TNF-α disrupted the mitochondrial membrane potential, induced ROS production, stimulated Drp1 phosphorylation and cytochrome c release, and activated the pro-apoptotic pathway. These observations are consistent with the data obtained in vivo from the HFD-fed mouse kidney and confirm our speculation that hyperglycemia, hyperlipidemia, and chronic inflammation are the key pathogenic factors to induce kidney injury under HFD feeding. We also demonstrated that depletion of ROS with a ROS scavenger ameliorated the detrimental effects of HG, PA, and TNF-α in both HK-2 and mesangial cells. Moreover, we also confirmed that the depletion of ROS with NAC was able to attenuate oxidative stress, prevent mitochondrial dysfunction, and block cytochrome c-mediated apoptotic pathways in the kidney cortex. Together these observations suggest that oxidative stress caused by nutrient overload plays a critical role in driving the molecular events leading to renal cell injury. Indeed, mitochondrial dysfunction produces ROS and vice versa, and this mutual promotion between mitochondrial dysfunction and ROS production appears to create a positive feedforward loop to drive renal cell injury. NAC is known to have activities beyond anti-oxidative stress such as anti-inflammatory actions via inhibiting NF-κB^28^, but whether these non-anti-oxidant activities are also involved in the protection of renal cells remains to be determined.

The Western lifestyle with diets rich in saturated fats is believed to be a major driving factor for the development of the obesity pandemic around the globe, and now obesity has been recognized as an important risk factor for the development of CKD. This study identifies mitochondrial dysfunction as a key pathogenic event in the development of kidney injury in a HFD environment. Therefore, in addition to careful control of dietary contents or components, molecular events leading to ROS production and mitochondrial dysfunction might also be used as therapeutic targets for the prevention of CKD.

Finally, this work has a number of limitations. Although our data suggest that mitochondrial dysfunction is a major driver for kidney injury, we did not directly count the mitochondrial number or measure the fission process. Moreover, the parameters used to define kidney injury in our mouse model may be different from those used clinically, and the experimental HFD used in the study is not exact the same as the high-fat-containing diets or Western diets consumed by humans. These limitations may need to be kept in mind when assessing the conclusion.

## Materials and methods

### Animals and treatment

Six-week-old male C57BL/6 mice were housed with a 12 h/12 h light/dark cycle and randomly allocated to two groups. One group was fed a low-fat diet (LFD, TP23524, Trophic Diet, China), which derives 10% calories from fat and 70% calories from carbohydrate, and another group was fed a HFD (TP23520, Trophic Diet, China), which derives 60% calories from fat and 20% calories from carbohydrate. Male mice were selected for this study as they were more sensitive to HFD induction than female mice in terms of the development of obesity and kidney injury. During 16 weeks of dietary treatment, body weight and blood glucose levels were monitored biweekly and every 4 weeks, respectively. At the end of the 16-week period, GTT, and ITT were performed. Kidneys were collected immediately after euthanasia for histological and biochemical analyses. All animal studies were approved by the Institutional Ethical Committee of China Medical University.

### Blood and urine analyses

Blood and urine samples were collected from the mice in the morning after 12-h fasting. Urinary albumin was quantified using an ELISA kit from Bethyl Laboratories, as described^29^. Urinary creatinine, serum BUN, serum creatinine, plasma FFA, plasma cholesterol, and plasma triglyceride were determined separately using commercial assay kits from Nanjing Jiancheng Bioengineering Institute (Nanjing, China) according to the manufacturer’s instructions.

### Glucose tolerance test (GTT)

After overnight fasting, mice have intraperitoneally injected glucose at 1.0 g/kg, and the change of blood glucose was monitored by a glucometer using tail blood collected at 0, 15, 30, 60, and 120 min after injection.

### ITT

Mice were administered human insulin (Wangbang Biopharmaceuticals, China) at 0.75 U/kg by intraperitoneal injection, and the change of blood glucose was measured at 0, 15, 30, and 60 min after insulin injection using tail blood.

### Histological analysis

Freshly harvested kidneys were cut in halves and fixed in 4% formaldehyde (pH 7.2), embedded in paraffin, and sectioned at a thickness of 4 μm. Hematoxylin and eosin (H&E) staining and Periodic acid-Schiff (PAS) staining were performed as previously described^29,30^. Cell apoptosis was determined by TUNEL staining using a commercial TUNEL kit from Roche (USA).

### Kidney lipid extraction and analysis

Lipids were extracted from the kidney cortex according to a rapid lipid extraction method described by Bligh and Dyer^31^. The extracted lipids were naturally dried and weighed. Triglyceride and cholesterol contents were quantified using commercial assay kits purchased from Nanjing Jiancheng Bioengineering Institute.

### Transmission electron microscopy

Kidney tissues were cut into small pieces and fixed in 2.5% glutaraldehyde for 12 h, and then post-fixed in 1% osmium tetroxide. Fixed sections were dehydrated through graded alcohols, penetrated with acetone, and embedded in epoxy resin. Ultrathin pieces (70–90 nm) were stained with uranyl acetate and lead citrate, and examined under an electron microscope (Hitachi, Tokyo, Japan).

### Cell culture

HK-2 cells (CRL-2190, ATCC) and SV40 MES 13 mesangial cells (MC) (CRL-1927, ATCC) were routinely cultured in DMEM/F12 supplemented with 10% fetal bovine serum (FBS) at 37 °C and 5% CO_2_. These cells were mycoplasma negative. Before treatment, the cells were synchronized in serum-free DMEM overnight, and then changed to DMEM containing 5 mM glucose (LG), 25 mM glucose (HG), 100 nM palmitic acid (PA), or 30 ng/mL TNF-α, or various combinations of these factors for 24 h. In some experiments, the cells were pre-treated with 5 mM N-Acetyl-L-cysteine (NAC, A9165, Sigma-Aldrich) for 1 h before these treatments.

### Ex vivo kidney tissue culture

Ex vivo kidney tissue culture was performed according to previously published procedure^7^. Briefly, the kidney cortex was cutoff from freshly harvested kidneys from mice treated with HFD or LFD for 16 weeks, and the cortex tissues were cut into ~70 μm^3^ pieces before being cultured in DMEM/F12 supplemented with 10% FBS at 37 °C and 5% CO_2_ in the presence or absence of 5 mM NAC for 8–12 h. Following the treatment, the tissue pieces were collected for the measurement of ROS production and western blot analysis of proteins.

### Mitochondrial membrane potential and mitochondrial distribution

Mitochondrial membrane potential (△Ψm) was assessed using a JC-1 Mitochondrial Membrane Potential Assay kit (C2006, Beyotime, China) as described previously^32^ following the manufacturer’s protocol. Flow cytometry was performed in a BD LSRFortessa flow cytometer. Intracellular distribution of mitochondria in HK-2 cells were labeled with MitoTracker Red CMXRos (Beyotime, China) in culture media at 37 °C for 30 min and then observed by laser-scanning confocal microscopy.

### Mitochondrial and cytoplasmic fractionation

Mitochondrial and cytosolic fractionation was performed using a Mitochondria Isolation kit (Beyotime, China) according to the manufacturer’s instructions. Briefly, fresh kidney samples or HK-2 cells were washed once with PBS, placed in mitochondria extraction mixed buffer provided in the kit, and then homogenized using an ice-cold Dounce tissue grinder. The homogenates were centrifuged at 600×*g* for 10 min, and the supernatant was further centrifuged at 11,000×*g* for 10 min at 4 °C. The supernatant was collected, and the precipitate consisted of the mitochondria. Cytosolic proteins were isolated from the supernatant following further centrifugation at 12,000 ×*g* for 10 min at 4 °C. The mitochondrial participates were dissolved in the mitochondria lysis mixed buffer to obtain mitochondrial proteins. Cytochrome c release from mitochondria was evaluated by western blot analysis of cytosolic proteins.

### Assessment of ROS

Intracellular ROS generation was quantified with a ROS-sensitive fluorescent probe 2’,7’-dichlorodihydrofluorescein diacetate (DCFH-DA) using a Reactive Oxygen Species Assay kit (S0033, Beyotime, China), as described previously^32^. HK-2 cells following various treatments or single-cell suspensions prepared from fresh kidney tissues were incubated with 10 μM DCFH-DA at 37 °C in the dark for 20 min. After three washes with PBS, the cells were observed under a fluorescence microscope (Leica, Germany).

### Western blot

Western blot analyses were carried out as described previously^33^. The following antibodies were used: TGF-β1 (ab92486, Abcam), α-SMA (23779489, Millipore), collagen type I (ab21286, Abcam), ACL (ab40793, Abcam), HMGCR (ab174830, Abcam), DGAT1 (ab54037, Abcam), Gp91 (ab129068, Abcam), TNF-α (ab9739, Abcam), Caspase-3 (9662, Cell Signaling), PUMA (ab9643, Abcam), Cytochrome c (ab133504, Abcam), Drp1 (5391, Cell Signaling), and phospho-Drp1(Ser616) (4494, Cell Signaling).

### Quantitative RT-PCR

Total RNAs were extracted using TRIzol reagent (Invitrogen). First-strand cDNA templates were synthesized using PrimeScript RT reagent kit (TaKaRa, Mountain View, CA). Real-time PCR was carried out using SYBR Premix Ex kit (TaKaRa) in a ROCHE 96 real-time PCR system. Relative amounts of transcripts were calculated using the 2^-∆∆Ct^ formula as described^34^, using GAPDH as an internal control. PCR primers are provided in Table 1.

**Table. 1:** Nucleotide sequences of PCR primers used in the study.

Primer	Forward (5′–3′)	Reverse (5′–3′)
mDGAT1	CTGTGCTCATGTATGTCCACGACT	CTGGCTCATACCAGTGATGAGATT
mDGAT2	GGAGCCGCAAAGGATTTGTA	AATAGGTGGGAACCAGATCAGC
mMOGAT1	TGGTGCCAGTTTGGTTCCAG	TGCTCTGAGGTCGGGTTC
mMOGAT2	TGGGAGCGCAGGTTACAGA	CAGGTGGCATACAGGACAGA
mIL-6	ATAGTCCTTCCTACCCCAATTTCC	CTGACCACAGTGAGGAATGTCCAC
mLeptin	CCAAAACCCTCATCAAGACC	GTCCAACTGTTGAAGAATGTCCCC
mTNF-α	TCAGCCTCTTCTCATTCCTG	CAGGCTTGTCACTCGAATTT
mMCP-1	CAAGAAGGAATGGGTCCAGA	TGAGGTGGTTGTGGAAAAGG
mIL-1β	GAAATGCCACCTTTTGACAGTG	TGGATGCTCTCATCAGGACAG
mα-Actinin-4	GCCATCCAGGACATCTCTGT	CCGCAGCTTGTCATACTCAA
mCD2AP	AGGAATTCAGCCACATCCAC	TTGAGGGAAACAGTCCCAAC
mNephrin	CCCCTCTATGATGAAGTACAAATGGA	GTACGGATTTCCTCAGGTCTTCT
mPodocin	GTGTCCAAAGCCATCCAGTT	GTCTTTGTGCCTCAGCTTCC
mGAPDH	TGTGTCCGTCGTGGATCTGA	CCTGCTTCACCACCTTCTTGA

### Statistical analyses

Data values were expressed as mean ± SEM. Statistical analyses were carried out using unpaired two-tailed Student’s *t* test for two group comparisons, or one-way analysis of variance (ANOVA) for three or more group comparisons. *P* < 0.05 were considered as statistically significant. Most experiments were repeated with similar results. To avoid interexperimental variations in statistical analysis, only the statistical results from one representative experiment were presented. Investigators were not blinded during the experiment and outcome assessment.
